# Clinical Value of ^99^Tc^m^-MDP SPECT Bone Scintigraphy in the Diagnosis of Unilateral Condylar Hyperplasia

**DOI:** 10.1155/2014/256256

**Published:** 2014-05-11

**Authors:** Bing Wen, Ying Shen, Chang-Yin Wang

**Affiliations:** Department of Nuclear Medicine, Zhongnan Hospital of Wuhan University, 169 Donghu Road, Wuchang, Wuhan, Hubei 430071, China

## Abstract

*Purpose*. To investigate the clinical value of ^99^Tc^m^-MDP SPECT for the diagnosis of unilateral condylar hyperplasia (UCH). * Methods*. One hundred forty-nine patients who underwent mandibular ^99^Tc^m^-MDP SPECT between January 2009 and December 2012 were studied, including 105 cases that were clinically suspected of UCH and 44 comparable cases without UCH as a control group. * Results*. Increased bone activity was observed in the affected condyles for all UCH patients. In the UCH group, the relative percentage uptake on the affected side was 59% (SD ± 4.3%), significantly higher than the 41% (SD ± 4.1%) uptake on the contralateral side (P<0.001). Similarly, the condyle/skull ratio was significantly higher for the affected side (1.66 ± 0.63) than for the contralateral side (1.34 ± 0.34, *P* < 0.01. No significant difference was found in the control group between the left and right condyles. Values for the sensitivity (95%), specificity (61%), positive (84.4%) and negative (84.6%) predictive values, and accuracy (84.5%) for ^99^Tc^m^-MDP SPECT in the diagnosis of UCH were calculated. However, for the hyperplastic condyle, no correlation was observed between the thickness of each cartilage layer and the relative uptake in the SPECT image. * Conclusion*. ^99^Tc^m^-MDP SPECT is accurate for diagnosing UCH and can provide a reference for treatment options.

## 1. Introduction

Unilateral condylar hyperplasia (UCH) is a rare disease that manifests as nonneoplastic overgrowth of the unilateral mandibular condyle, without analogous pathologic change in any other joint. This disorder often results in facial asymmetry, deformity, and malocclusion and is sometimes associated with obvious pain and dysfunction. It seems that UCH is common and frequently occurs in groups during the growth phase, especially in adolescence [1]. Females also appear to be more sensitive to UCH than males, so sex may be considered a risk factor [2].

The asymmetry associated with UCH can be classified into three categories: hemimandibular hyperplasia, hemimandibular elongation, and a hybrid form. The first type is the result of unilateral growth in the vertical plane, leading to a sloping rima oris, with little or no deviation of the chin. Hemimandibular elongation is characterized by asymmetry in the transverse plane, resulting in deviation of the chin towards the contralateral side [3]. Appropriate therapeutic decisions can be made only after thoroughly assessing the facial asymmetry [4], which is mainly based on subjective clinical evaluation and is supported by bone scans [5, 6].

Although condylar hyperplasia is a self-limiting condition, it is characterized by progressive and independent growth that always results in the bone volume of one condyle being greater than that of the other. According to the growth state, condylar hyperplasia is divided into an active phase and stationary phase. The activity level of the condyle is considered to be highly correlated with mandibular asymmetry [7, 8]. When making a therapeutic plan, it is important to determine whether there is excessive condylar activity, for example, with the use of a skeletal scintigraphy [8], which has recently been used in the diagnosis of condylar hyperplasia. Routine imaging examinations, such as X-ray and CT, can only detect anatomical changes and not condylar growth activity. Single photon emission computed tomography (SPECT) has been commonly used to evaluate metabolism in several types of tissue [9–11]. Importantly, SPECT could provide both functional and morphological information associated with condylar hyperplasia [12]. By using a bone-seeking radiopharmaceutical agent, areas of increased osteoblastic activity can be highlighted so that condylar activity can be examined.

Because clinical and radiographic presentations in patients with condylar hyperplasia are highly diverse, the assessment of condylar growth activity has a vital role in managing these patients [13]. Radionuclide bone imaging is a useful quantitative tool for indicating continued bone activity, but there are still some problems with assessment and diagnosis [14]. The purpose of this paper is to explore the diagnostic value of ^99^Tc^m^-MDP (technetium methylene diphosphonate) SPECT in UCH.

## 2. Materials and Methods

### 2.1. Patient Recruitment

In total, 105 patients with suspected UCH were observed in this study, including 42 males and 63 females aged 13–33 years, with an average of 21.5 years. As previously described [15], all suspected UCH patients underwent routine clinical examination, and plaster models and plain radiographs were taken to look for signs of condylar hyperplasia, including vertical, transverse, or vertical and transverse plane facial asymmetry with matching occlusal changes. Each patient underwent ^99^Tc^m^-MDP SPECT of the temporomandibular joint (TMJ) between January 2009 and December 2012, and 58 of the patients accepted high condylectomies and pathological examinations. The demographic and clinical characteristics of these patients are shown in Table 1. A control group of 44 age- and gender-matched patients (22 males and 22 females, 17 to 26 years' old, mean 22.6 years' old) underwent whole body scans using ^99^Tc^m^-MDP SPECT because of other diseases such as malignancy, and specific tomography of the TMJ was performed after informed consent. To minimize the influence of unnecessary interfering factors on ^99^Tc^m^-MDP uptake in the TMJ, the control group was limited to patients with complete dentition and without skeletal metastasis, malocclusion, facial tumor, facial trauma, or TMJ disorder.

### 2.2. SPECT Bone Imaging

Three to six hours after the injection of 740–925 MBq ^99^Tc^m^-MDP via elbow veins, SPECT bone images were obtained with an E.CAM SPECT scanner (Germany, Siemens). By using high-resolution, low-energy collimators, the SPECT scanner collected data over 360° in a spherical field of rotation, using a 128 × 128 matrix at 15 seconds per frame. The data from each case were reconstructed and reformatted into coronal, sagittal, and axial planes.

The SPECT images were analyzed by 2 experienced nuclear medicine physicians; the bone metabolism of each TMJ was routinely elevated by visual inspection, and a region of interest (ROI) method was applied. ROIs were drawn around each TMJ that represented the area of prominent ^99^Tc^m^-MDP uptake. To ensure that the left- and right-sided ROIs were of comparable size, the ROI was copied to the contralateral condylar region. The ratio of the unilateral count to the gross count of both sides was calculated for relative uptake [14]; 55% or above was considered an indication of a hyperactive condyle. Furthermore, an ROI was also set over the parietal bone as a control, and the ratio of each TMJ ROI count to the parietal ROI count was calculated.

### 2.3. Pathologic Analysis

The surgically removed condylar tissues were fixed by 10% paraformaldehyde, then embedded in paraffin, and serially cut for hematoxylin-eosin (HE) staining. Two experienced pathologists performed the pathological analysis independently from the results of the bone scan. The thickness of perichondrium, proliferation layer, and hypertrophic cartilage layer were measured under light microscope. Condyles in active growth were characterized by obvious cell hyperplasia, a thickened proliferative zone and hypertrophic layer, which were not observed in condyles in a stationary phase.

### 2.4. Statistical Analysis

The statistical analysis software SPSS version 16.0 (SPSS Inc., Chicago, IL) was used, and values were represented as the mean ± standard deviation (SD). Comparisons of ROI counts were performed using Student's *t*-test. The Pearson correlation test was used to determine the correlation between the relative uptake of affected condyles and the thickness of each cartilage layer. A *P* value of less than 0.05 was considered significant.

## 3. Results

### 3.1. SPECT Imaging Analysis

**Figure 1:**
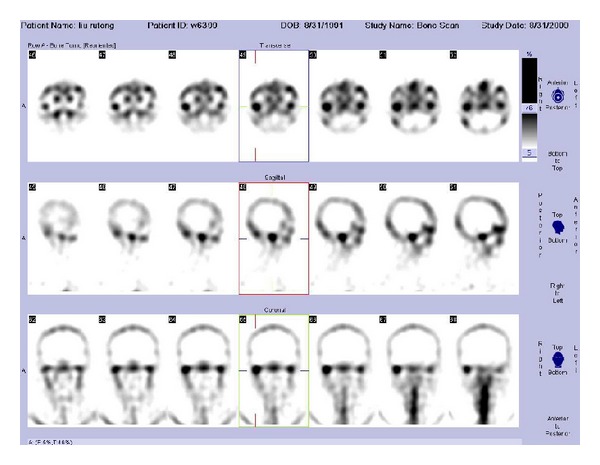
TMJ SPECT images of a patient (male, 21-year old) with right-sided condylar hyperplasia. An abnormally high uptake of radioactivity in the right condyle was observed in the axial, sagittal, and coronal reconstructions relative to the left side.

**Figure 2:**
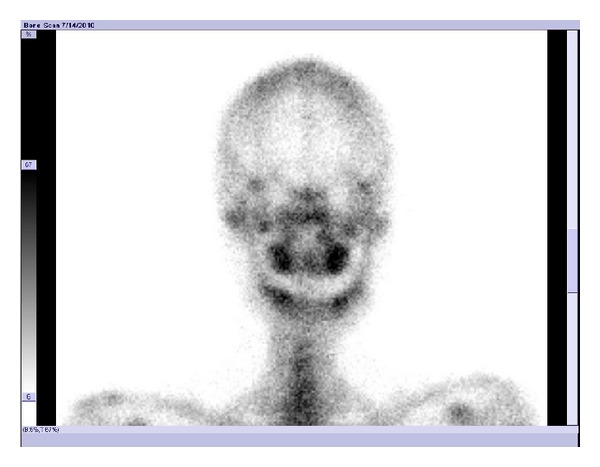
Anterior planar image of the head of a patient (male, 17-year old) with right side condylar hyperplasia. The center of the mandible was biased towards the left, and the bone metabolism of the right condyle was found to be active.

The control group had uniform and symmetrical radioactivity distribution on both sides of the TMJ based on visual inspection. However, for patients with UCH, the activity in the affected condylar region was obviously higher than that in contralateral condylar region, supported by a relatively concentrated area of radioactivity on the affected side (Figure 1). In anterior planar images of head, a shift of the mandible from the affected side to the contralateral side was observed (Figure 2).

Quantitative analysis of ROIs revealed differences in condylar activity (Table 2). The mean relative percentage uptake of the suspected hyperplastic condyle in the UCH group was 59% (SD ± 4.3%) and was significantly higher than that of the unaffected condyle (41%, SD ± 4.1%) (*t* = 6.590, *P* < 0.001). As for the ratio between the condyle and the skull, the values of the affected and unaffected condyle were also significantly different at 1.66 (SD ± 0.63) and 1.34 (SD ± 0.34) (*t* = 3.687, *P* < 0.01).

However, no significant difference was found between the left and right condyles in the control group. The uptake percentages of the left and right condyles were similar at 50% (SD ± 1.7%) and 50% (SD ± 6.2%) (*t* = 0.762, *P* > 0.05). The condyle/skull ratios were 1.12 (SD ± 0.07) and 1.10 (SD ± 0.06), also not significantly different (*t* = 1.832, *P* > 0.05).

The UCH group and the control group were also compared. The mean relative percentage uptake was significantly higher in the affected condyles of the suspected UCH patients than in the left condyles of controls (*t* = 4.750, *P* < 0.001). Similarly, the condyle/skull ratio was significantly higher for the affected condyles of the suspected UCH patients than for right condyles of the controls (*t* = 6.459, *P* < 0.001).

### 3.2. ^99^Tc^m^-MDP SPECT Imaging for Assessment of Condylar Growth

Visual and quantitative analysis of ^99^Tc^m^-MDP SPECT were available for all 105 patients. Seventy-three cases showed increased radioactivity on the affected side (relative uptake ≥ 55%); 45 of them underwent condylectomy, and active growth was pathologically confirmed in 38 cases. To prevent deformity recurrence, 13 of the remaining 32 cases that presented no activity in the SPECT images also underwent condylectomy; pathologic analyses confirmed that 11 cases were in the stationary phases of growth (Table 3). The efficacy of ^99^Tc^m^-MDP SPECT imaging for evaluating condylar active growth was characterized by a sensitivity of 95% (38/40), specificity of 61.1% (11/18), positive predictive value of 84.4% (38/45), negative predictive value of 84.6% (11/13), and accuracy of 84.5% (49/58).

### 3.3. Pathological Features of Condylar Hyperplasia

**Figure 3:**
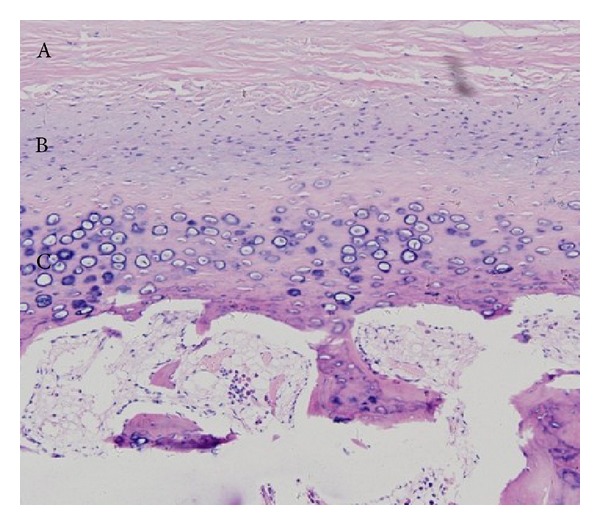
The pathology image of a patient (male, 21-year old) with UCH (HE ×100). (A) Obvious thickened perichondrium; (B) wide proliferative zone, small and flat-shaped undifferentiated mesenchymal cells distributed sparsely; (C) thickened hypertrophic layer (calcified cartilage layer).

The histopathologic evaluation of the resected condyles showed that the condylar cartilage was divided into four layers: the perichondrium, proliferative zone, hypertrophic layer, and calcified cartilage layer. Condylar cartilage with active growth showed an obviously thicker proliferative zone and hypertrophic layer, accompanied by remarkable hyperplasia of undifferentiated mesenchymal cells from the proliferation layer and hyaline chondrocytes from the hypertrophic layer; periosteal thickening was observed in some cases (Figure 3). The perichondrium of condylar cartilage in a stationary phase was visibly thickened, without cell hyperplasia of proliferative and hypertrophic layers. Seven cases with positive SPECT imaging results were recognized as stationary by pathology, with no excessive chondrocyte growth but visible, aspecific periosteal thickening. One case returned negative SPECT results, though the hyaline chondrocytes of the hypertrophic layer were observed to actively proliferate. The relative percentage uptake of the affected condyles was compared to the corresponding pathological results. The relative uptake value was significantly higher in patients with a remarkable hyperplasia of mesenchymal cells and hyaline chondrocytes than in patients without remarkable hyperplasia or with a mild hyperplasia (67.4 ± 8.2% versus 58.8 ± 3.5%, *P* < 0.01). The values for subjects in the stationary phase did not exceed 0.55. Condylar hyperplasia was defined as excessive growth of condylar cartilage, with thickening of the perichondrium, proliferative zone, and hypertrophic layer. No correlation was found between the relative percentage uptake and mean thickness of these layers in the 45 patients of the UCH group who underwent condylectomy and histological analysis (*r* = 0.46, 0.47, −0.12, resp., *P* > 0.05).

## 4. Discussion

In the present study, the role of ^99^Tc^m^-MDP SPECT in diagnosing UCH was evaluated. Our results found SPECT to be an accurate method for recognizing hyperactivity in a condyle, through analysis of the relative percentage uptake of ^99^Tc^m^-MDP, with a cutoff value of 55%. Increased radionuclide uptake in the hyperplastic condylar region can constitute evidence of continued abnormal growth.

Currently, it is impossible to define an absolute age of presentation for UCH [1, 2, 16]. The patients who participated in this study were between 13 and 33 years of age, with a mean age of 21.5, which is consistent with previous reports that UCH usually affects teenagers and young adults between the ages of 10 and 30 [1, 16, 17]. Although some reports have found no relationship between sex and UCH incidence, Raijmakers et al. [2] proposed that women were at greater risk, with an incidence of 64%. This gender dependence was confirmed in this study, with a male-to-female ratio 1 : 1.5 in the 105 cases. No differences were detected in UCH incidence between the left and right condyle in our study, consistent with the previous study [1].

Radionuclide bone imaging has a unique advantage in evaluating the ongoing activity of condyle. ^99^Tc^m^-MDP SPECT of the TMJ can clearly show whether the bone metabolism is overactive or not. A relative uptake of at least 55% was regarded as an indication of hyperactive condyles, as this value distinguishes actively growing condyles from stationary ones [14, 15]. Using this value, a high sensitivity of 95% was reached for the 58 cases of condylar hyperplasia according to pathological examination; however, the specificity was relatively low (61.1%). These data differ slightly from the results of some earlier studies [18–20], which reported a higher value of specificity; differences in statistical methodology may be one cause of this discrepancy. Additionally, the evaluation criterion is an important factor because there is no generally accepted gold standard for diagnosing UCH. Our study used histopathological analysis as a reference standard, but some previous studies have used clinical performance instead. Interobserver variability during drawing and placement of the ROI can also affect the variability of results.

Currently, surgery followed by orthodontic operation is the main treatment for condylar hyperplasia. Optimal treatment planning always depends on precise assessment of the condylar growth state. An active mandibular condyle with a related, progressing facial deformity often requires high partial condylectomy to limit the growth of the condyle. Additionally, to prevent recurrence, surgery to correct any residual deformity should be performed when the excessive growth of condyle has ceased [21]. Conversely, an inactive condyle can be maintained and directly treated with a corrective operation through the osteotomy of the upper and lower jaws to correct occlusal and facial abnormalities.

A typical SPECT image of the hyperactive condyle in this study is characterized by an abnormally high uptake of ^99^Tc^m^-MDP. Therefore, when the relative uptake exceeds 55%, dynamic observation combined with evaluation of clinical characteristics is recommended. Orthognathic surgery can be performed directly in cases without any progressing facial deformities. In cases with a progressing facial deformity, a longer follow-up of 6 to 12 months is necessary, and it is suggested that a SPECT examination be performed again after 6 months. If the relative uptake is less than 55%, only orthognathic surgery is required. Conversely, if the relative uptake remains above 55%, a partial condylectomy should be performed. If the result of the initial SPECT scan shows that the relative uptake is less than 55%, direct orthognathic treatment can be provided.

Condylar cartilage can be divided into 4 layers according to surface depth: the perichondrium, proliferative zone, hypertrophic layer, and calcified cartilage layer. The pathological changes of condylar hyperplasia are mainly characterized by obvious thickening of the cartilage layer [22, 23]. Variation in the cartilage layer may be associated with excess bone formation and simultaneously affected by other variables [24]. In this study, active condylar cartilage was characterized by the remarkable proliferation of undifferentiated mesenchymal cells in the proliferative zone and hyaline chondrocytes in the hypertrophic layer. The perichondrium, proliferative zone, and hypertrophic layer were also observed to thicken to some extent. Although thickened perichondrium was found in the inactive condyle, no excessive growth of cells was detected. The relationship between histopathological changes and condylar activity has been explored using various approaches. Villanueva-Alcojol et al. [16] categorized condylar hyperplasia of 36 cases into 4 histopathologically different types, and no correlation was found between the SPECT results and the pathological type. This result is consistent with many reports that have found no typical histologic patterns in patients with UCH [22, 24]. Farina et al. [23] analyzed 8 cases of condylar hyperplasia and also found no correlation between radioactivity uptake in the SPECT image and the pathological manifestation. However, they were able to observe a positive correlation between the thickness of the condylar cartilage and the number of granules in argentums nucleolar organizer regions in young patients. Theoretically, inactive cartilage remnants can be reactivated, which may be the underlying mechanism for the development of condylar hyperactivity [25]. Our study showed that the relative uptake of the affected condyle is not related to the thickness of cartilage layers but is affected by the proliferation of undifferentiated mesenchymal cells and hyaline chondrocytes. In active condyles with obvious hyperplasia of undifferentiated mesenchymal cells, almost all values of the relative uptake were above 0.60. Therefore, the high uptake of radioactivity as observed in the condylar SPECT image may indicate hyperplasia of condylar cartilage cells.

## 5. Conclusion

Our results suggest that ^99^Tc^m^-MDP SPECT is important for the assessment of condylar activity. It is an accurate method for identifying UCH patients with ongoing bone growth, and it is helpful to not only for the diagnosis of condylar hyperplasia but also for choosing an appropriate therapy.

## Figures and Tables

**Table 1:** Clinical characteristics of the 105 patients diagnosed with UCH.

Female, *n* (%)	63 (60)
Age, mean (range)	21.5 (13–33)
Mean duration of symptoms (years)	5.3 (0.5–11)
Main complaint, *n* (%)	
Asymmetry	68 (64.7)
Pain and dysfunction	29 (27.6)
Asymmetry, pain, and dysfunction	8 (7.6)
Affected joint (right/left bilateral), *n* (%)	
Female	29 (46.0)/34 (54.0)
Male	23 (54.8)/19 (45.2)

**Table 2:** Quantitative analysis of the ROI in patients with UCH and controls.

	UCH group (SD)		*P* value	Control group (SD)		*P *value
	Affected	Contralateral		Right	Left	
Relative uptake (%)	59 (4.3)	41 (4.1)	<0.001	50 (1.7)	50 (6.2)	>0.05
Condyle/skull	1.66 (0.63)	1.34 (0.34)	<0.01	1.12 (0.07)	1.1 (0.06)	>0.05

**Table 3:** SPECT and pathological results.

	Positive SPECT	Negative SPECT	Total
Positive pathology	38	2	40
Negative pathology	7	11	18

Total	45	13	58
